# Modular multi-channel high voltage arbitrary waveform generator and imaging setup for dielectric elastomer actuator characterisation

**DOI:** 10.1016/j.ohx.2024.e00526

**Published:** 2024-04-02

**Authors:** M. Vu, M. Lewandowski, X. Guo, A. Weightman, S. Watson, T.J. Echtermeyer

**Affiliations:** a Photon Science Institute, University of Manchester, Oxford Road, Manchester M13 9PL, United Kingdom; b Department of Electrical and Electronic Engineering, University of Manchester, Oxford Road, Manchester M13 9PL, United Kingdom; c Department of Computer Science, University of Manchester, Oxford Road, Manchester M13 9PL, United Kingdom; d Department of Mechanical Aerospace and Civil Engineering, University of Manchester, Oxford Road, Manchester M13 9PL, United Kingdom; e National Graphene Institute, University of Manchester, Oxford Road, Manchester M13 9PL, United Kingdom

**Keywords:** DEA, Multi-channel, High voltage, Arbitrary waveform, Arduino, DAQ

## Abstract

Various applications require multi-channel high-voltage sources for their control, e.g. electrostatic adhesion, electrophoresis and artificial muscles such as piezoelectric, hydraulically amplified self-healing electrostatic(HASEL) and dielectric elastomer actuators(DEAs). Further, the ability to simultaneously monitor the state of the actuators either with images, or voltage and current sensing is crucial to characterise their behaviour. In this work, we present the design of a versatile characterisation setup, capable of generating eight HV (15 kV) arbitrary waveforms(rise time of 8 ms and fall time of 80 ms for 60 MΩ load), while synchronously monitoring voltage and current, and record high-speed (120 fps) video. The setup ensures modularity and customisability by consisting of three independent modules: (1) The imaging module includes a Raspberry Pi and a Pi Camera; (2) A 3.3 V analogue interface 16-bit resolution data acquisition module on a PCB that accommodates a microcontroller board, two 8-channel analogue-to-digital converters, and an 8-channel digital-to-analogue converter; (3) Up to 8 DC-to-HVDC converter boards powered by 12 V DC, with 3.3 V analogue interface.

## Graphical abstract

**Figure:**
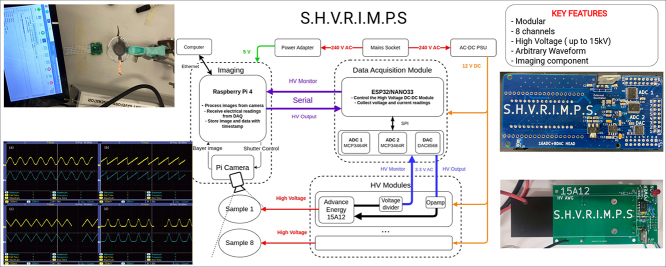

## Specifications table

**Table:** 

**Hardware name**	SHVRIMPS: Synchronised High-Voltage aRbItrary Modular Power Supply

**Subject area**	Engineering and material science

**Hardware type**	Electrical engineering and computer science

**Closest commercial analogue**	Commercial programmable HV generators/HV amplifier

**Open source license**	CC BY-SA 4.0

**Cost of hardware**	•£440 for the bare bone high voltage module.
	•£620 for the single channel with imaging.
	•£3700 for the 8-channel setup.

**Source file repository**	https://OSF.IO/YTCDV

	

## 1 Hardware in context

Dielectric elastomer actuators (DEAs) and hydraulically amplified self-healing electrostatic (HASEL) actuators are emerging as potential biomimetic structures, mimicking muscle-like movements with comparable energy densities, actuation strain, and power output across a broad frequency range, from sub-Hz to hundreds of Hz [1], [2], [3], [4]. DEAs are comprised of compressible dielectric membranes, typically acrylic or silicone-based and ranging from 10–100 μm in thickness, sandwiched between compliant electrodes. The application of a high voltage/potential difference between the electrodes (in the order of several hundred to a few thousand Volts) induces electrostatic attraction, resulting in Maxwell pressure on the dielectric membrane. This pressure compresses the membrane in thickness while expanding its lateral dimensions.

The main challenge in operating DEAs is the high voltage requirement, often in the range of thousands of Volts for elastomers, corresponding to an electric field of approximately 100 $V\;\mu m^{-1}$ [4], [5], [6], [7]. Especially in applications like medical orthoses, soft robotics, and haptic devices, multiple DEAs are commonly arranged to work together, necessitating different driving signals of multiple time-synchronised high-voltage arbitrary waveforms to stimulate the actuators. The future development of DEAs and their integration into applications requires a versatile power supply capable of generating arbitrary multi-channel high-voltage signals, along with simultaneous, synchronised data acquisition capabilities to monitor properties and states of the DEA, such as charge/current flow, potential differences, and physical deformation through imaging, all at an affordable cost.

Currently, commercial all-in-one solutions offering these capabilities are nonexistent. Partial solutions involving separate imaging and high-voltage components are expensive, not easily accessible, and often have long lead times. USB plug-and-play high-speed cameras with hardware triggers, like Luxonis OAK-D [8], Venus VEN-301-125U3C-FPC [9], and ThorLab CS135 [10], range from £200($249) to £1500($1727) but still require a dedicated computer for interfacing with high-voltage components.

High-voltage supplies with single outputs have drawbacks, such as DC-only output, lack of monitoring capabilities, or discontinuation [11]. Using several multiple single-output power supplies increases complexity when synchronising their outputs. Multi-channel power supplies from Caen [12] or Physical Instruments [13] cost over £10 000($12 000).

Beyond proprietary solutions, there are two open-hardware power supplies with high-voltage and multi-channel output: Peta-Pico-Voltron (PPV) [11] and a pocket-sized ten-channel design [14]. Both use a fixed HV rail combined with HV-optocouplers to isolate low and high voltages. The PPV, while capable of producing high slew rate 1 kHz HV square waves, cannot generate arbitrary waveform signals. The pocket-sized design addresses this limitation by pulse-modulating optocouplers to charge and discharge the actuator, incorporating a PID controller for feedback. Although unable to produce arbitrary waveforms, this setup allows actuators to be held at arbitrary potential levels, with response time dependent on PID controller settings. While cost and space-efficient, obtaining high-voltage optocouplers remains challenging and costly, requiring custom orders with lead times of around 14 weeks.

To address these challenges, we present SHVRIMPS: Synchronised High-Voltage aRbItrary Modular Power Supply. SHVRIMPS is modular, utilising standard off-the-shelf components, and easily adaptable for various applications. It comprises three main components: (1) a Raspberry Pi 4b-based imaging system with a camera for live video streaming, (2) a DAQ board compatible with easily programmable microcontrollers (e.g., Arduino Nano 33 BLE [15] or ESP32 DevKitC [16]), and (3) a multi-channel arbitrary waveform HV supply.

To overcome the above shortcomings we designed SHVRIMPS: Synchronised High-Voltage aRbItrary Modular Power Supply. SHVRIMPS is modular, consisting of standard, off-the-shelf components, and easy to modify for various applications. It consists of three main components: (1) an imaging system based on a Raspberry Pi 4B with a camera, producing a live video stream (2) a DAQ board compatible with easily programmable microcontrollers, such as Arduino Nano 33 BLE [15] or ESP32 DevKitC [16], and (c) a multi-channel arbitrary waveform HV supply.

## 2 Hardware description

### 2.1 Overview

SHVRIMPS is a high-voltage generator and optical imaging setup, capable of

• Outputting arbitrary high-voltage waveforms with an amplitude of 15 kV and a rise time less than 10 ms for load resistances below 8 MΩ.
• Eight independent synchronous and asynchronous 16-bit output channels.
• Monitoring the output high voltages and currents at adjustable sampling rate. Maximum rate of 9875 samples per second.
• Recording video of samples at a rate of 120 fps at 640 × 480 pixels.
• Displaying live video feed, control panel, and monitoring data on a GUI, accessible over Ethernet.

**Fig. 1:**
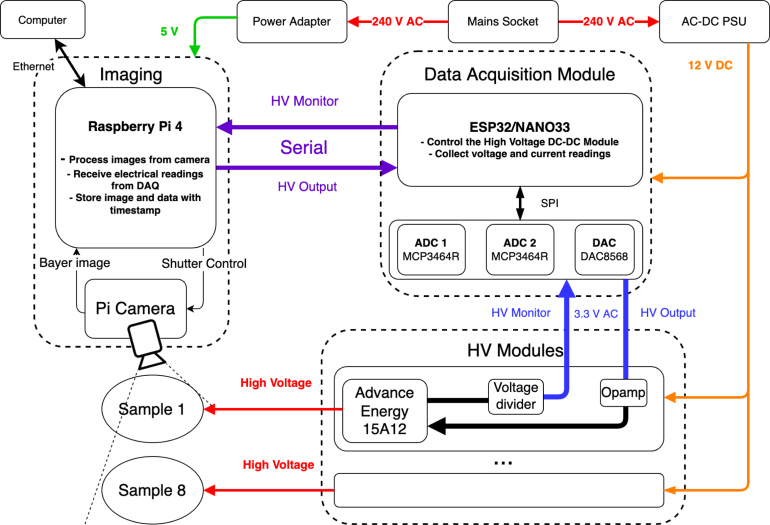
Block diagram of the SHVRIMPS: The imaging block is top left. The DAQ module is top right. The high voltage module is bottom right.

In Fig. 1, the operational setup comprises three subsystems: (1) Imaging, (2) Data Acquisition (DAQ), and (3) High Voltage (HV). Functioning akin to a real shrimp, a collaborative effort between a camera and a Raspberry Pi serves as the *‘eyes’* and *‘brain’*, capturing videos while establishing communication with the DAQ via USB Serial. Serving as the *‘ventral nerve cord’* of SHVRIMPS, the DAQ incorporates a microcontroller and Analogue/Digital interfaces (including Analogue-to-Digital Converter (ADC) and Digital-to-Analogue Converter (DAC)). The DAQ utilises a 3.3 V AC signal to manage the HV module (*‘appendages’*), while simultaneously coordinating with the imaging module for *‘appendages-eye-coordination’*. The high voltage output is employed to activate the DEAs, and their responses are captured by the camera.

The data acquisition board design prioritises simplicity and minimalism, achieved through the use of a small number of easily assembled components. We advocate for the modularity of our design, facilitating straightforward customisation, component replacement, testing, debugging, and future upgrades. As an example, the DAQ board can be substituted with a commercial alternative like the PicoLog 1000 Series [17] to streamline assembly. All components within the subsystems are easily accessible, owing to their widespread popularity and availability from various electronic suppliers. Design considerations include the potential substitution of certain subsystem components, such as the microcontroller, ADC, DAC, and operational amplifiers, with alternatives to minimise build time in the event of component unavailability, with details outlined in the Bill of Materials.

For software control and coding simplicity, we opted for widely used microcontrollers, namely the Arduino Nano 33 BLE [15] and ESP32 DevKitC [16]. The imaging module is based on a Raspberry Pi 4B with a Pi Camera v2, chosen for its cost-effectiveness (approximately £91/$115) and capability to achieve framerates of at least 100 fps [18]. Embracing open access principles, the entire project was developed using popular open-source and free software tools, including KiCAD for PCB design [19], the Arduino IDE for microcontroller firmware [20], and Python for programming the Pi [21]. In summary, the simplicity, modularity, and accessibility of our setup are designed to facilitate easy adaptation to specific needs and functionalities.

**Fig. 2:**
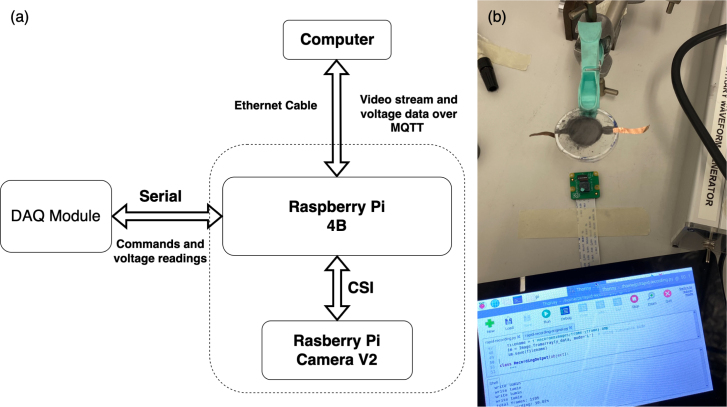
Imaging Module: (a) Block diagram showing the internal communication of the module (the Pi and the camera) and the external communication (Pi to DAQ and PC). (b) Raspberry Pi (with LCD) and camera measuring the areal expansion of a DEA.

### 2.2 Imaging module

The imaging module utilises the Raspberry Pi 4 for its programming simplicity, accessibility, and extensive camera compatibility [18]. Leveraging the capable onboard processor and graphics computing unit, the Raspberry Pi can capture, process, and store image streams at 640 × 480 pixels in YUV420 format, achieving a frame rate of 120 fps. Concurrent with image acquisition, the Raspberry Pi establishes USB communication with the DAQ board, retrieving output data from the HV modules. It also timestamps the output voltages and currents, associating them with the current frame in the video (see Fig. 2(a)). An illustrative setup is depicted in Fig. 2(b), where the camera is positioned on a table to record videos of a suspended DEA sample. The presented setup allows users to observe the stretch of the membrane when a specific voltage is applied. Through straightforward video processing techniques, the setup enables the automatic collection of a substantial dataset for characterising the effects of input signal frequency, amplitude, and waveforms such as sine, square, sawtooth, etc. Moreover, the high fps, combined with the time stamping, facilitates the measuring of the transient mechanical response under electrical excitation of DEAs.

The Raspberry Pi’s Ethernet port facilitates rapid and low-latency video transmission. For data transmission, the widely adopted MQTT protocol [22] is employed, utilising an Eclipse Mosquitto server [23]. A computer linked to the Pi via an Ethernet cable gains access to the web-based GUI, which displays the video stream, voltage monitoring data, and the control for the HV output (Fig. 7. Beyond streamlining data collection, the Pi and camera afford a degree of separation between the user and high-voltage elements, allowing for operation from a safe distance. To further enhance high-voltage isolation, the Ethernet signal can be converted to fibreoptics communication using a modem.

### 2.3 Data Acquisition Module (DAQ)

**Fig. 3:**
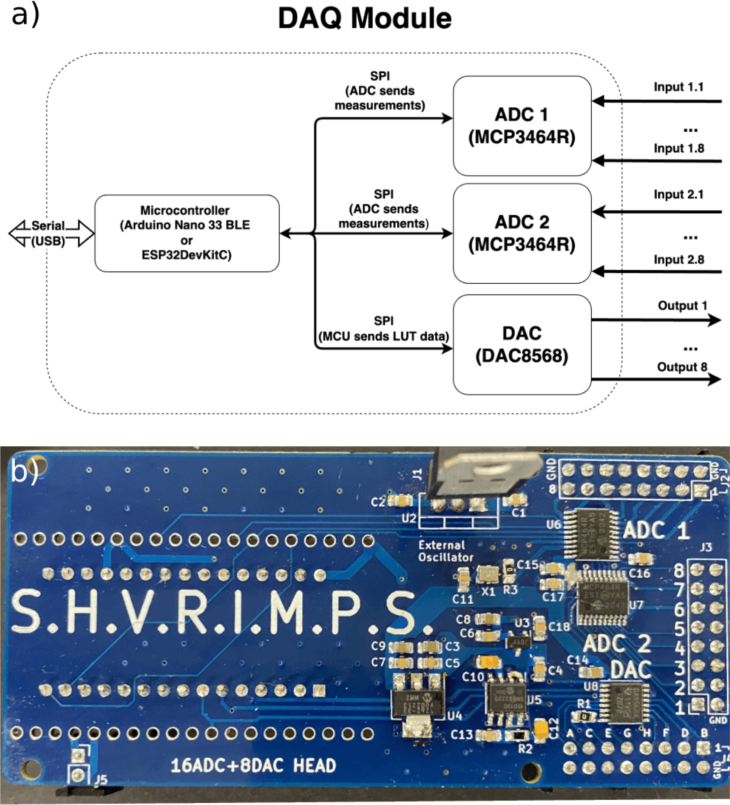
Data Acquisition Module: (a) Diagram showing the internal and external communication. The physical location of the internal blocks approximately matches the position of the physical PCB below. (b) Soldered DAQ Module.

The DAQ module functions similar to a hardware CODEC, converting analogue signals to digital and vice versa over multiple channels. This method is used to control and monitor the HV modules. Fig. 3a shows the block diagram of the module. The DAQ houses one microcontroller board (Arduino Nano 33 BLE or ESP32 DevKitC) that communicates with two ADC and DAC ICs over SPI to code and decode analogue signals in 16-bit resolution. The arbitrary waveform is represented as a normalised lookup table, an array of float values in the range [0, 1]. The float values are then converted to 16-bit unsigned values, and then transmitted over SPI to the DAC. To achieve a specific output frequency, the time steps between the data points can be set, and the microcontroller then uses this timing to wait between outputting the DAC values. The analogue voltages produced by the DAC are then transmitted to the HV module where they are amplified to HV signals. The voltage and current outputs of the HV modules (V${}_{mon}$, I${}_{mon}$) are fed back to the ADC, converted to digital values and transmitted by the microcontroller over USB Serial to the Raspberry Pi for storage and visualisation.

Fig. 3b shows an image of the assembled DAQ PCB. An Arduino Nano 33 BLE has been soldered on the right underside of the board (underneath the SHVRIMPS text). On the right, the ADCs and DAC are marked in large white texts next to their header pins; marked A to H for the DAC, and 1–8 for the ADCs. In the middle, from top to bottom, is the 12-to-5V linear regulator (LM7805/U2), a clock signal generator (ECS-2520/X1), 5-to-3.3 V linear regulator (MCP1754/U3), 5-to-3.3 V linear regulator (MCP1825/U4), and precision voltage reference (MCP1501/U5). For more details on the physical layout of the PCB, please refer to Table 2 or the design files in the board1_Arduino_DAC_ADC/* folder of the repository.

The ADC (MCP3464R) and DAC (DAC8568) chips were selected for their capacity for 8-channel operation and provision of 16-bit resolution, translating to 50 μV per least significant bit (LSB) with a 3.3 V reference, coupled with minimal noise. Notably, the DAC chip offers synchronous outputs, facilitating effortless synchronisation of the output channels. Communication occurs through the standard SPI protocol, ensuring high-speed data transfer. The MCP3464R and DAC8568 can be interchangeably substituted with alternative ICs from the same family (refer to Table 2 and the detailed Bill of Materials), featuring diverse resolutions and channel counts. Importantly, ICs within this family share identical pinouts, simplifying substitution in cases of scarcity, cost adjustments, or performance customisation.

### 2.4 High voltage module

**Fig. 4:**
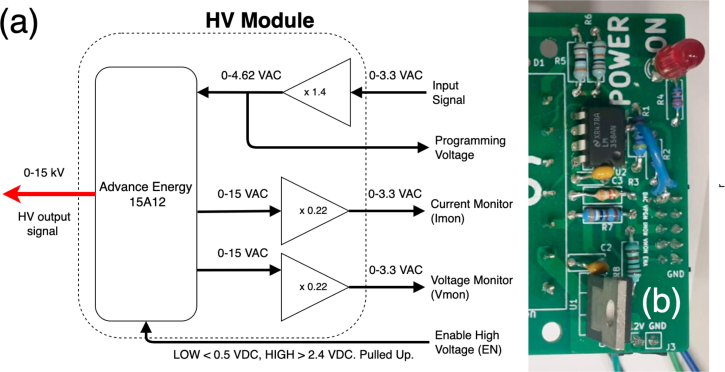
High Voltage Module: (a) Diagram showing the constituent blocks and the internal and external communication. (b) A populated HV module PCB.

A DC-HVDC converter (Advance Energy 15A12) forms the basis of the HV module, with its block diagram shown in Fig. 4. The converter can provide up to 15 kV output voltage and can linearly be controlled in the range from 0–15 kV by a programming voltage from 0–4.64 V. It measures 4 cm × 12 cm and can be PCB mounted. The module has a specified ramp-up time of 150 ms at 100% load, equivalent to 100 k Vs^−1^ [24], which is verified and further characterised in Section 7. The module’s high voltage and low current output is monitored and regulated internally, and can also be externally read-out with signals ranging from 0–15 V, corresponding to the minimum and maximum levels, respectively. The module requires a supply voltage in the range of 11–16 V. The non-uniformity of the voltage levels of these various supply, control and feedback signals is mitigated by a custom PCB that facilitates the interaction with the DC-HVDC module. The board has five communications pins which output and accept any 3.3 V signal, a standard voltage level for low-power electronics. The input signal controlling the HV output is amplified by a single supply operational amplifier (LM358/U2) with a non-inverting gain factor of 1.4 to achieve the aforementioned 4.64 V programming range (Fig. 4(a). The voltage and current monitor is connected to a voltage divider to scale signals from 15 V down to 3.3 V. Two more header pins are reserved for the 12 V power and ground pins.

Further components on the board include a LED (D2) as a visual indicator for when the HV-HVDC converter is powered and the Zener diode (D1, not visible in the figure) as voltage surge protection. A linear voltage regulator (LM7809/U1) reduces the 12 V supply voltage down to 9 V to supply the LM358.

### 2.5 Versatility

SHVRIMPS holds versatile potential for a range of applications. Besides the straightforward modification to control the HV source without the imaging component, the data acquisition module is suitable as a data logger for use cases such as a high-resolution, multi-channel sensor controller or as an expansion board for embedded applications. Further, since the DAQ module has been designed to accommodate two popular microcontrollers, researchers from other fields can easily modify the presented system according to their needs.

The HV modules utilised in this project are capable of generating up to 15 kV with 4 W of power. Beyond its application in DEAs, the high-voltage system can find utility in various domains, including capillary electrophoresis [25], drivers for piezoelectric actuators, mass spectrometry, electron microscopes, and technologies such as plasma and cathode ray tubes (CRT) [24].

## 3 Design files summary

**Table:** 

Design filename	File type	Open source license	Location of the file
hardware/ board1_Arduino_DAC_ADC/*	Schematics, PCB, KiCad	CC BY-SA 4.0	available on repository

hardware/ board2_HV_MODULE_15A12/*	Schematics, PCB, KiCad	CC BY-SA 4.0	available on repository

software/client-software/*	Python, flask, GUI	CC BY-SA 4.0	available on repository

software/pi-software/*	Python, video streaming, MQTT setup	CC BY-SA 4.0	available on repository

software/SHVRIMS_arduino_code/*	Arduino Nano 33 BLE Code	CC BY-SA 4.0	available on repository

setup/*	Markdown, instructions	CC BY-SA 4.0	available on repository

1. hardware/board1_Arduino_DAC_ADC/*: the KiCad design files for the DAQ board.
2. hardware/board2_HV_MODULE_15A12/*: the KiCad design files for the High Voltage Module.
3. software/client-software/*: web-based GUI for the video feedback system, plotting voltages and timestamping (in setup, ran on laptop connected via ethernet cable to raspberry pi)
4. software/pi-software/*: the video streaming to MQTT python script and reading voltage monitoring data over serial streaming to MQTT python script
5. software/SHVRIMS_arduino_code/*: Arduino framework code (PlatformIO) for Arduino Nano 33 BLE
6. setup/*: various markdown files, with instructions how to setup connection, installation etc.

## 4 Bill of materials summary

This section shows a shortened bill of materials for the major components required to construct SHVRIMPS. The exhaustive bill of materials can be found in the repository hardware folder. The Excel file contains separate sheets for the DAQ and HV module, the power circuit and consumables (see Table 1, Table 2, Table 3).

**Table 1:** Bill of materials for the imaging module.

Ref	Qnty	Value	Description	Manufacture number	Alternative option	Unit price	Total price
	1		Raspberry Pi 4B with MicroSD card and power supply		Jetson Nano	£65 ($82)	£65 ($82)
	1		Pi Camera v2		Pi Camera v3	£26 ($33)	£26($33)

					**Total**		**£91 ($116)**

**Table 2:** Bill of materials for the DAQ module.

Ref	Qnty	Value	Description	Manufacture number	Alternative option	Unit price	Total price
A1,	1	Arduino_Nano_v3.x	Arduino Nano 33 BLE	ABX00030	ESP32-DevKitC V4	£25.86 ($33)	£25.86 ($33)

U2,	1	LM7805_TO220	Positive 1 A 35 V linear regulator, Fixed output 5 V, TO-220-3	L7805ABV		£0.72 ($0.92)	£0.72 ($0.92)

U3,	1	MCP1754S-3302xCB	Fixed 150 mA low dropout voltage regulator, Positive, 3.3 V output, SOT-23	MCP1754ST-3302E/CB	LDO with the same specification and footprint (make note of the pin layout as different manufacturers have different ordering).	£0.45 ($0.57)	£0.45 ($0.57)

U4,	1	MCP1825S-3.3V	500 mA, Low-voltage, Low quiescent current LDO regulator, SOT-223-3	MCP1825ST-3302E/DB	LDO with the same specification and footprint (make note of the pin layout as different manufacturers have different ordering).	£0.58 ($0.74)	£0.58 ($0.74)

U5,	1	MCP1501-33xSN	3.3V, 0.1%, 20 mA, Precision voltage reference, SOIC-8	MCP1501-30E/SN	LDO with the same specification and footprint (make note of the pin layout as different manufacturers have different ordering).	£1.29 ($1.64)	£1.29 ($1.64)

U6, U7,	2	MCP3464R	TSSOP-20_4.4 × 6.5 mm_P0.65 mm	MCP3464R-E/ST	MCP3x6x family	£4.38 ($5.57)	£8.76 ($11.14)

U8,	1	DAC8568IAPW	TSSOP-16-	DAC8568IAPW	DAC856x family	£21.65 ($27.53)	£21.65 ($27.53)

X1,	1	ECS-2520	HCMOS crystal clock oscillator, 2.5 × 2.0 mm SMD	ECS-2520MVLC-049-BN-TR	Oscillator with frequency under 20 MHz and with similar footprint.	£1.17 ($1.49)	£1.17 ($1.49)

	1	PCB	4-Layer PCB			£1.31 ($1.67)	£1.31 ($1.67)

Cx, Rx, Dx, Jx		Passive components	Capacitors, Resistors, Diodes and Pin headers				£27.40 ($34.84)

						**Total**	**£89.19** ($113)

**Table 3:** Bill of materials for the HV module.

Ref	Qnty	Value	Description	Manufacture number	Alternative option	Unit price	Total price
U1,	1	LM7809_TO220	Positive 1 A 35 V linear regulator, Fixed output 9 V, TO-220-3	L7809ABV		£0.655 ($0.83)	£0.655 ($0.83)

U2,	1	LM358	Dual low-noise operational amplifiers, DIP-8	LM358AP	Other single supply dual Op Amps	£0.353 ($0.45)	£0.353 ($0.45)

U3,	1	15A12P-4	High voltage DC-DC module			£433 ($550)	£433 ($550)

	1	PCB	2-Layer PCB			£2 ($2.5)	£2 ($2.5)

Cx, Rx, Dx, Jx		Passive components	Capacitors, Resistors, Diodes and Pin headers				£7.64 ($9.7)

						Total	£444 ($565)

						**Total for 8**	**£3550 ($4514)**

## 5 Build instructions

### 5.1 DAQ module

Most of the components for the DAQ module are surface mount (SM) packages, so the recommended assembly is to use solder paste (we have used CHIPQUIK SMD291SN) and either a standard PCB oven or hot plate for surface mounting. Alternatively, some PCB manufacturers (JLCPCB) provide services for soldering and assembling complete boards with surface-mounted components. Finally, although difficult, hand soldering is also possible.

**A. Using Oven or Hot plate**

**Fig. 5:**
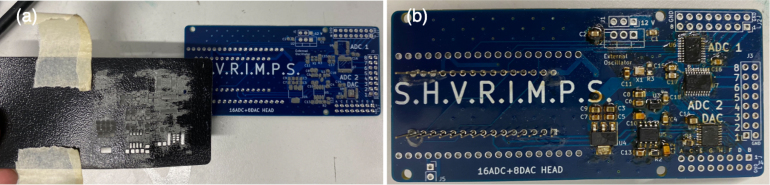
(a) DAQ module with solder paste applied. (b) DAQ module after hot plate.

1. Order a stencil together with the PCB from the PCB manufacturer.
2. Using the stencil, apply solder paste on the pads, scraping off any excess, leaving a small amount behind. Fig. 5(a).
3. Place the SM device components on the pads. Through-hole (TH) components will be soldered manually later in a subsequent step. Be mindful of the orientation of the SM components, particularly the ADC and DAC components as their orientation is not clearly indicated. Refer to KiCad files from ‘hardware/board1_Arduino_DAC_ADC/*’, the labels on the solder mask, and Bill of Materials to find the location and orientation of each component.
4. Put the board into the oven or place it on the hot plate for the reflow process. Set up the heating profile recommended by the solder paste manufacturer. An example of a finished board is shown in Fig. 5(b).
5. Inspect the board visually and with a multimeter to check for any shorts and damages to traces or solder mask. A good practice is to start from the 12 V pin(J1) and follow the power traces to 5 V, 3.3 V, and continuing until ending at the header pins connected to the ADC and the DAC. Any imperfections, unsoldered joints, or shorts can be cleared up by going through the reflow process again or by using a fine-tip soldering iron and/or solder wick.
6. Place and solder the remaining TH component with a soldering iron. The microcontrollers are located on the underside of the board, as marked on the silkscreen.
7. Repeat the inspection process of the board once more.
8. Use isopropyl alcohol and a brush to clean up flux residue if needed.

**B. Soldering by hand**

1. Solder the components using a soldering iron with a very fine tip and very fine soldering wire (≈0.2 mm wire) for small components. Refer to KiCad files from ‘hardware/board1_Arduino_DAC_ADC/*’, the labels on the solder mask, and Bill of Materials to find the location of each component.
2. Assembly is easiest when starting with the smaller components, e.g. starting with the smallest packages like ADC(U6, U7) and DAC(U8), then the capacitors, resistors and the power ICs and then moving on to the large TH elements. The most difficult components are the MCP3464 (ADC) and the DAC8568 (DAC) due to their small pitch size. A good approach is to solder the pins with as little solder as possible and clean up any short or excess with a solder wick.
3. Inspect the board visually and with a multimeter to check for any shorts and damages to traces or solder mask. A good practice is to start from the 12 V pin(J1) and follow the power traces to 5 V, 3.3 V, and continuing until ending at the header pins connected to the ADC and the DAC. Any imperfections, unsoldered joints, or shorts can be cleared up by going through the reflow process again or by using a fine-tip soldering iron and/or solder wick.
4. Clean up flux residue with a brush, paper towels and isopropyl alcohol.

### 5.2 HV module(s)

The HV module was designed for ease of assembly. All the components are through-hole which can be soldered with a standard soldering iron.

1. Solder the components in the order of ascending vertical height, starting with ‘low-profile’ components like the resistors and diodes then ending with the header pins and the HV module. Refer to KiCad files from ‘hardware/board2_HV_MODULE_ 15A12/*’ for the location of each components.
2. Inspect the board visually and with a multi-meter, checking for any shorts and damages to traces or solder mask.
3. Repeat for as many boards as necessary.

**Fig. 6:**
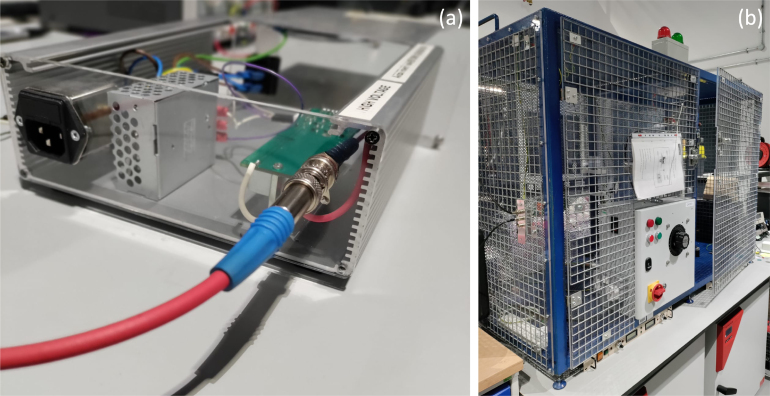
(a) Panel mounted SHV connector with a high-voltage coaxial cable connected. (b) High-voltage test cage.

### 5.3 Assembly

Once the PCBs for the DAQ board and the HV module(s) have been populated, they need to be connected.

1. Make sure that when wiring, the modules are not powered.
2. Connect the DAQ board to the Raspberry Pi or laptop with a USB cable using the USB port on the microcontroller.
3. Connect the HV modules: start by firstly connecting the (DAC Input signal/3.3 V Input Signal (labelled as DAC pin on board) to the DAQ input signal) and ground pins on the HV module and DAQ together
4. Connect the V${}_{mon}$ and I${}_{mon}$ from the HV module to the DAQ board (the ADC channels).
5. Connect the load (DEA) to the HV cable and HV ground return cable.
6. With the 12 V power supply off, plug in the power supply cables to the HV modules.
7. Turn on the power supply. The LED present on the HV modules should light up to indicate the high-voltage module is powered.

For more detailed information about the setup, please refer to the repository.

### 5.4 Enclosures

Given the setup’s high-voltage capabilities, there is a significant risk of arcing, which poses a hazard to users. To enhance safety, it is advised that the setup be operated within a confined space to prevent unintended contact with high-voltage components (Fig. 6(b). If enclosing the setup is impractical, at the very least, it should be contained within a grounded metallic enclosure. The high-voltage output should be linked to an SHV connector (we have used RADIALL R317580000). Externally, a coaxial HV cable (hivolt HRG58-20-2) could be attached to an SHV jack (RADIALL R317072000) on one side, with the opposite end directed towards the experiment area away from the user (Fig. 6(a)).

## 6 Operation instructions

### 6.1 Graphical user interface

**Fig. 7:**
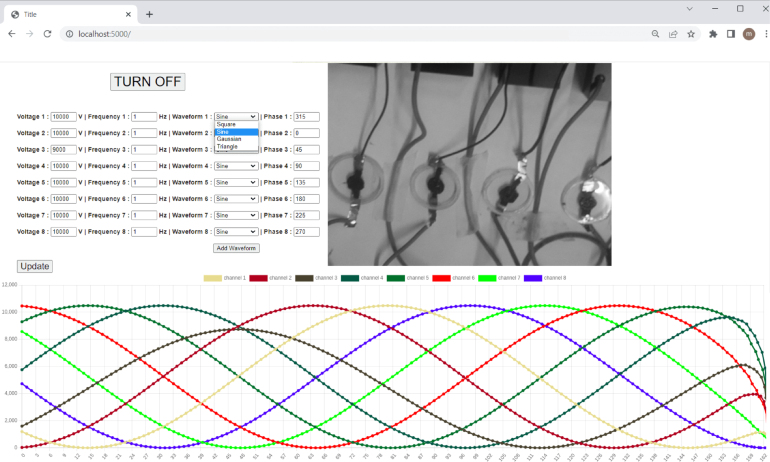
GUI viewed on a web browser. The controls to set the voltage, frequency, wave shape, and phase shift are at the top left. The centre shows a live camera view. The bottom shows a moving window of the output voltages of the 8-channels of the HV module.

1. Plug in the power to the Raspberry Pi 4B (needs to have enabled SSH and Pi camera in configuration, and laptop/PC needs to have ‘shared to other computer’ enabled for the Ethernet connection.)
2. Plug the Pi camera into the CSI port of the Raspberry Pi.
3. Plug the Ethernet cable into the port on the Pi and laptop/PC.
4. SSH to Raspberry Pi using terminal command ssh pi@raspberry.local.
5. Transfer the files available from repository (the software/pi-software/* folder) to raspberry pi.
6. In order to install and run MQTT server, run install.sh, install python dependencies by running pip install -r requirements.txt from software/pi-software/* folder
7. Run python MQTT services by typing in python3 videoStreaming.py and python3 dataStream.py commands on the terminal.
8. On PC/laptop install python3 and install dependencies required for running GUI (pip install -r requirements.txt from software/client-software/* folder).
9. Start the GUI by running python3 main.py (from software/client-software/* folder).
10. Navigate to web browser, go to localhost:5000 address to see the GUI. Fig. 7 shows an example of the GUI, displaying the output controls, the video stream and live view of eight sinusoidal, phase-offset HV signals.

### 6.2 High voltage setup

Despite the modules having a relatively low power output of 4 W, it is imperative for researchers who are attempting to replicate the setup to adhere strictly to all the safety precautions taken when working with high voltage. Authoritative guidelines can be found on NIST’s EEEL Safety Rules for Moderate and High Voltages [26] and the IEEE Standard for High-Voltage Testing Techniques (IEEE Std 4™-2013) [27]. Below are some essential safety points, along with our recommendations:

• Maintain a minimum distance of 50 mm between any object with bare conductors at ground potential and high-voltage points.
• Verify that all connections, including HV probes, grounds, and electrodes, are secured and reliably connected.
• If feasible, encase the entire apparatus in a separate enclosure to prevent inadvertent contact with high-voltage electrodes. Emergency power-off switches should be installed at a safe distance from the setup for quick access. We have used three distinct switches: one to control the 12 V input to the module, one to control the power to the entire unit, and one to disconnect the mains power.
• Display clear warning indicators, such as red “HIGH VOLTAGE” signs, in the vicinity of the setup to alert those unfamiliar with its operation to the potential risks.
• Given that the setup operates at high voltages, where currents may surpass a few milliamperes, it is advisable to adopt a buddy system for safety. Always work with a partner and avoid leaving the equipment operational without supervision.

## 7 Validation and characterisation

### 7.1 Data acquisition and control signal precision

**Fig. 8:**
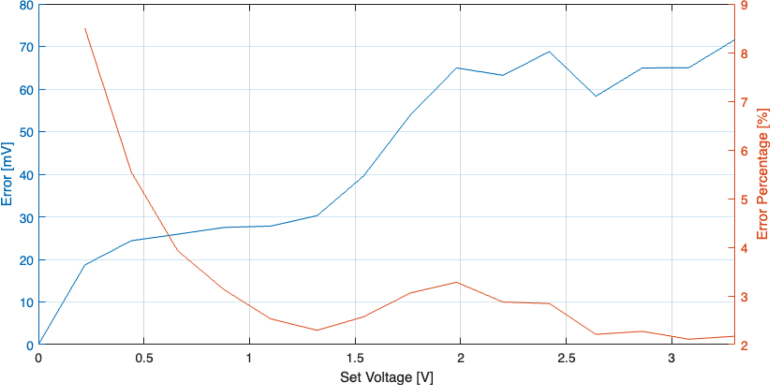
Absolute (blue) and relative (orange) error of the output signal produced by the DAC. (For interpretation of the references to colour in this figure legend, the reader is referred to the web version of this article.)

As the signal generated by the DAC is used to directly control the high voltage output, any error present would also be amplified. To validate the performance of the DAC, its output signal, used to program the HV module, has been varied from 0 to 3.3 V with 15 equal steps. This corresponds to a HV signal from 0 to 15 kV after amplification, with 1000 V increments. The DAC has been programmed to the discrete voltage levels and the output voltage is measured with an oscilloscope alongside their corresponding HV output to determine their accuracy. Fig. 8 shows the absolute and relative error of the DAC output. The largest error is approximately 70 mV, which is roughly 322 V on the high voltage side, at 15 kV, this is the equivalent of 2%.

**Fig. 9:**
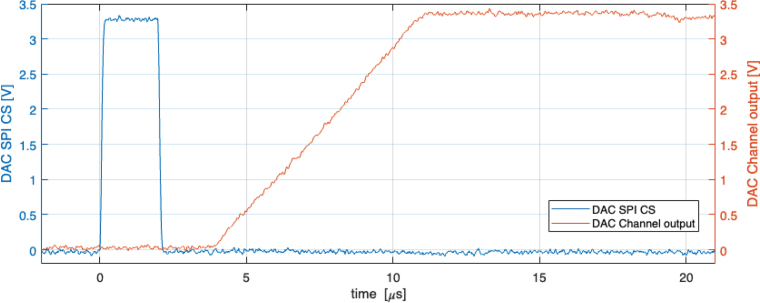
Delay between the SPI control signal (blue) and the DAC output voltage ramp up (orange). (For interpretation of the references to colour in this figure legend, the reader is referred to the web version of this article.)

The rate at which SHVRIMPS generates high voltages consists of two discrete timings, the rate at which the DAC respond to new configuration settings and the rise time of the HV module. The response speed of the DAC has been characterised by sending an SPI message to the DAC and recording the delay time of the DAC output. Fig. 9 shows the timing of the SPI message and DAC response, indicating a 9.1μs delay between the end of the SPI message and the DAC output achieving the full range output of 3.3 V. Given that the rise time of the HV module is in the order of ≈10 ms, the delay of the DAC is negligible and does not place any bottleneck on the overall performance of the setup. The response time of SHVRIMPS can therefore be considered to be equal to the response time of the HV module.

**Fig. 10:**
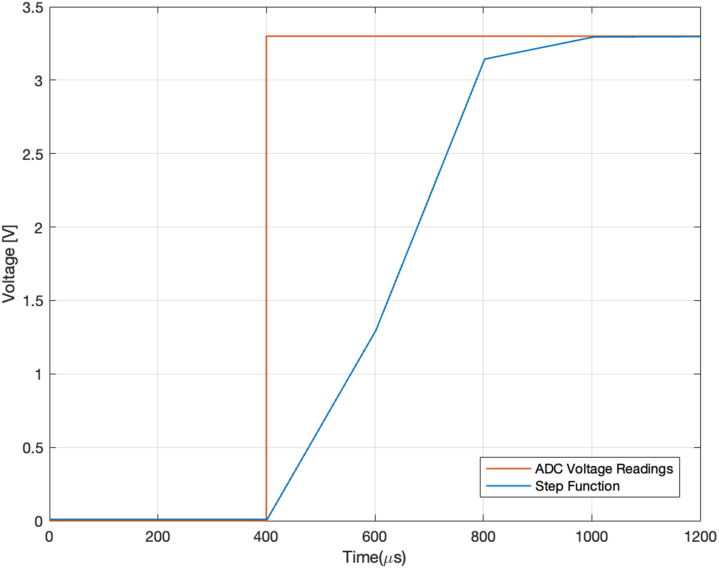
ADC’s reading(blue) of a step signal(orange).

Similarly, the sampling rate and sampling accuracy of the ADCs are important for monitoring the voltages and currents of the output. As per the datasheet [28], at an oversampling ratio of 256, the ADC has a sampling rate of 4800 Hz, equivalent to approximately 200μs per reading. Using this polling rate to measure a 3.3 V step function (rise time = 9.5 ns) shows a rise time of approximately 400μs. This is adequate for reading the output of the HV Module (discussed further below) and also other applications with ms timing (see Fig. 10).

**Fig. 11:**
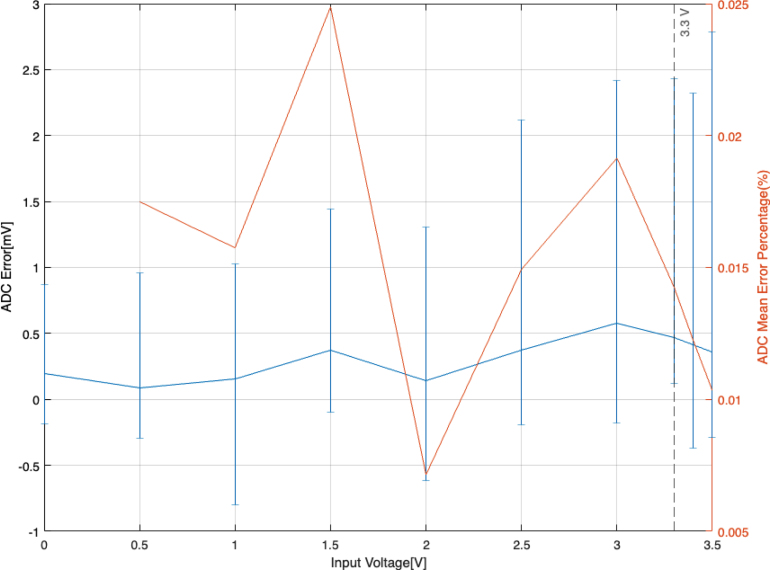
Absolute (blue) and relative (orange) error of the 30 measurements sampled by the ADC. (For interpretation of the references to colour in this figure legend, the reader is referred to the web version of this article.)

The accuracy and linearity of the ADC have been characterised by using it to measure the output voltage of a calibrated Keithley 2400 SourceMeter. 30 consecutive readings of the output voltage of the Keithley SourceMeter were performed, each at an oversampling rate of 256. Fig. 11 shows the error of the voltage measured by the ADC vs. the applied voltage. Absolute errors are between −0.05 to 1.96 mV, corresponding to a maximum relative mean error of 0.025%. This error correlates to a maximum error of 9 V on the high voltage reading. As there is not a clear relationship between the input voltage and the error, we can assume that the HV error is approximately 9 V across the full range. However, this error of 9 V is below the specified output ripple of the 15A12 DC-DC converter of approximately 18 V, thus can be considered more than sufficient.

Overall, the DAQ module provides DAC output signals with an error of 3.5% on average and a delay of ≈10μs as well as an ADC input signal error of less than 0.025% with a delay of 400μs for the voltage ranges relevant for the control of DEAs.

### 7.2 High voltage generation

**Fig. 12:**
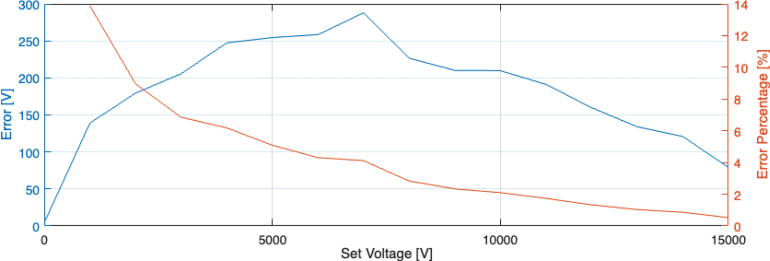
Absolute (blue) and relative (orange) error of the output signal produced by the HV output.

Knowing the capabilities and limitations of the DAQ, the HV output can now be characterised. Using the DAQ module to generate a rectangular function (step up and down), HV output voltages from 1000 V to 15 000 V with 1000 V increment have been programmed. A 60 MΩ load is fixed between the HV output and return. The HV signal response is then measured using a HV probe with a ratio of 2000:1. The deviation from the intended set voltage is shown in Fig. 12. The error decreases consistently towards larger voltages and is most severe for lower voltages. Particularly for lower voltages, the most feasible solution for mitigating the error is to tune the DAC output. This can be performed by either an open loop approach with a formula to adjust the output curve, or with a closed loop control algorithm like PID.

**Fig. 13:**
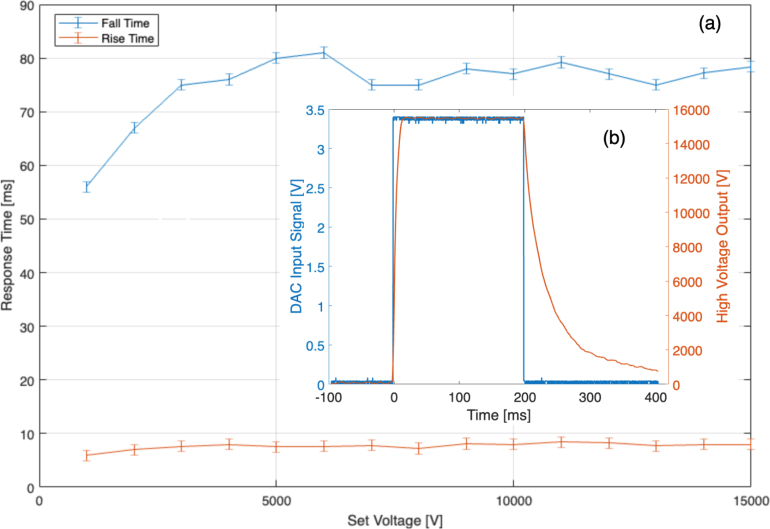
(a) Response time at specific voltage levels for a load impedance of 60 MΩ. (b) An example of a rectangular excitation signal (blue), and the corresponding HV response (orange). (For interpretation of the references to colour in this figure legend, the reader is referred to the web version of this article.)

A further important figure of merit is the time response of the HV signal. Fig. 13 shows the rise and fall time (10% to 90%, captured using an oscilloscope) of the HV signal for an outputted HV pulse between 0 V and various set voltages. It can be seen that the rise time is unaffected by the output voltage, consistently being within 7–8.4 ms. The fall time, however, is approximately an order of magnitude larger between 70 and 80 ms. Generally, both the rise and fall times are approximately constant over the complete output voltage range of the HV module.

**Fig. 14:**
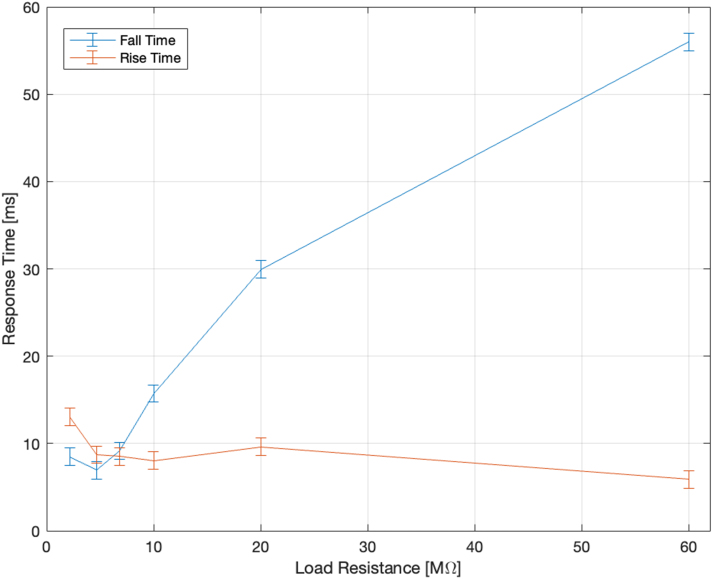
The effect of load impedance on the rise(blue) and fall(orange) time of a 1 kV rectangular signal.

Fig. 14 shows the effect of different load resistances on the rise and fall time of a 1 kV output signal. The rise time remains almost constant as a function of load resistance. The fall time increases significantly for load resistances ≫ 7 MΩ. Assuming the output resembles that of an RC low-pass filter, the 3 dB roll-off bandwidth is inversely proportional to the rise time, with a proportionality constant of 0.35. The operating frequency range for DEAs with resistances up to 7 MΩ is approximately $\frac{0.35}{risetime}=\frac{0.35}{0.00916}=38.89\;\mathrm{Hz}$, even 50 Hz at 5 MΩ. In general, the setup is able to drive DEAs with load resistances up to 60 MΩ at Hz and sub-Hz actuation frequencies.

### 7.3 Arbitrary and multi-channel synchronised high voltage generation

**Fig. 15:**
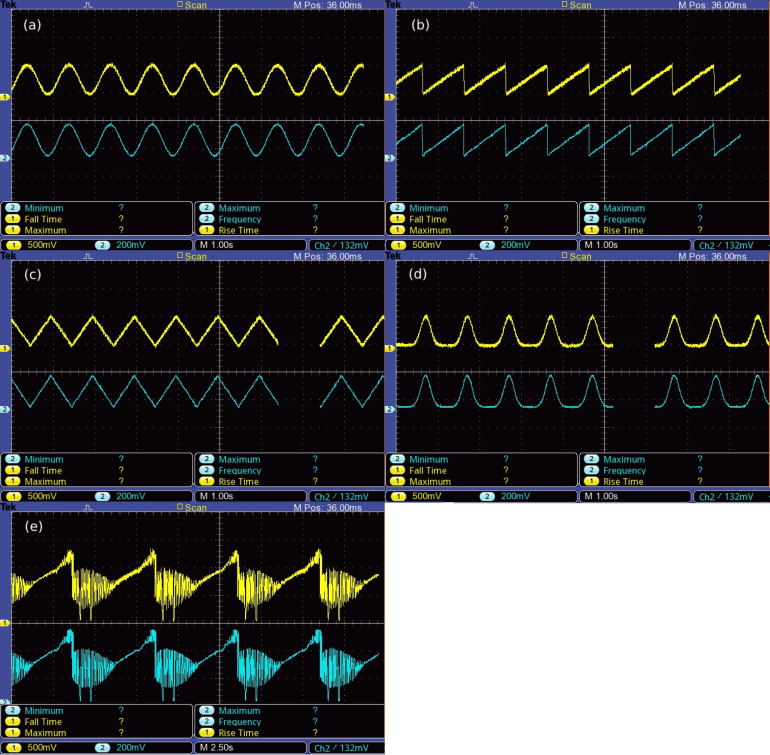
Exemplary waveforms produced with SHVRIMPS, probed with an oscilloscope. (a) Sine wave, (b) Sawtooth wave, (c) Triangle wave, (d) Gaussian, (e) Ducks. The yellow channel, at 500 mV per division, is the HV output connected to the scope via a HV probe with a ratio of 2000:1, i.e. each division corresponds to 1000 V output signal. The blue channel is the output signal from the DAQ module with 200 mV per division. Time base is 1 s/div, except for (e), which is 2.5 s/div. (For interpretation of the references to colour in this figure legend, the reader is referred to the web version of this article.)

As described in Section 2.3, SHVRIMPS can output arbitrary waveforms using look-up tables to set up waypoints. This capability is demonstrated in Fig. 15. Various waveforms such as sinusoidal, sawtooth, triangle, gaussian and ‘ducks’ [29] have been generated employing this method. Synchronous measurement of the DAQ output signal and the generated HV signal via a HV probe with an oscilloscope demonstrate the versatility of SHVRIMPS and the ability to generate arbitrary waveforms.

**Fig. 16:**
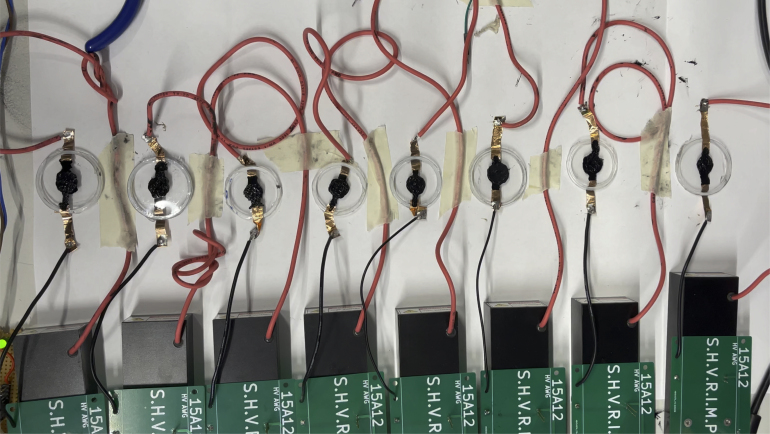
Example multi-channel setup driving 8 VHB DEAs.

A setup, Fig. 16, was assembled to demonstrate the independent control of eight classic DEAs made from VHB4905 tape, as described in [7]. First, eight synchronous sine waves were applied to the actuators to verify that there were no phase differences between them. Subsequently, 8 sine waves with a phase shift of $\frac{\pi }{4}$ were applied to the DEAs. The videos can be found in the repository folder ‘demonstration/videos’.

### 7.4 Summary

In summary, SHVRIMPS generates HV signals with an error below 14% over the HV range from 0 to 15 kV in an open-loop configuration. The output voltage response, specifically the fall time, is dependent on the load impedance. Rise and fall times below 10 ms are achieved for DEA load resistances below 8 MΩ. The specifications are shown in Table 4.

**Table 4:** Specifications summary of SHVRIMPS.

Parameters		Conditions	Value
Electrical	Maximum output voltage		15 kV
	Maximum output power		4 W
	Number of channel(s)		1–8
	Output signal waveform		Arbitrary
	Maximum rise time	15 kV 60 MΩ load	8 ms
	Maximum fall time		81 ms

Imaging	Maximum frame rate	At 640 × 480	120 fps
	Maximum resolution		3280 × 2464

## 8 Conclusions

SHVRIMPS emerges as a versatile platform engineered to generate arbitrary waveforms across eight independent channels, capable of driving DEAs within the sub-Hz to 50 Hz range at voltages up to 15 kV. The high-voltage output, with a rise time of approximately 10 ms and a fall time ranging from 55–80 ms, is well-suited for actuator control. The setup facilitates simultaneous monitoring of voltages and currents at the outputs while recording videos at a rate of 120 fps. Assembling the system is straightforward, utilising off-the-shelf components readily available from popular electronics retailers, with a total cost of £3700.

Future iterations of the project could enhance the DAQ board, addressing issues such as selecting superior ADC and DAC modules concerning noise, sampling frequency, and resolution. Refinements to the PCB, focusing on improved grounding, filtering, and layout for these ICs, aim to further elevate the system’s overall performance. Additionally, future iterations will focus on implementing galvanic isolation between the user and the HV module. While the existing setup allows remote access to the Raspberry Pi over Ethernet, the conversion of the Ethernet signal to fibreoptics communication stands out as a viable method to enhance isolation.

Recognising the importance of flexibility for researchers, we acknowledge that the frequency of the HV signal presents a limiting factor. In future iterations, the development of an HV module with a faster response is prioritised, enabling the creation of higher frequency, high-voltage signals and expanding the system’s applicability. Additionally, we are committed to exploring avenues to reduce the high cost associated with the off-the-shelf HV DC-DC converter component.

## CRediT authorship contribution statement

**M. Vu:** Conceptualization, Data curation, Methodology, Software, Visualization, Writing – original draft, Writing – review & editing. **M. Lewandowski:** Data curation, Investigation, Software, Visualization, Writing – original draft, Writing – review & editing. **X. Guo:** Resources. **A. Weightman:** Supervision. **S. Watson:** Supervision, Writing – review & editing. **T.J. Echtermeyer:** Funding acquisition, Investigation, Project administration, Supervision, Writing – review & editing.

## Declaration of competing interest

The authors declare that they have no known competing financial interests or personal relationships that could have appeared to influence the work reported in this paper.

## Appendix A Supplementary data

Video S1 SHVRIMPS outputing high voltage sine for 8 DEA actuators with 0 degrees phase shift between them.

Video S2 SHVRIMPS outputing high voltage sine for 8 DEA actuators with 45 degrees phase shift between them.
